# Industry in Motion: Using Smart Phones to Explore the Spatial Network of the Garment Industry in New York City

**DOI:** 10.1371/journal.pone.0086165

**Published:** 2014-02-05

**Authors:** Sarah Williams, Elizabeth Currid-Halkett

**Affiliations:** 1 Department of Urban Studies and Planning, Massachusetts Institute of Technology, Cambridge, Massachusetts, United States of America; 2 Sol Price School of Public Policy, University of Southern California, Los Angeles, California, Untied States of America; Instituto de Fisica Interdisciplinar y Sistemas Complejos IFISC (CSIC-UIB), Spain

## Abstract

Industrial agglomerations have long been thought to offer economic and social benefits to firms and people that are only captured by location within their specified geographies. Using the case study of New York City’s garment industry along with data acquired from cell phones and social media, this study set out to understand the discrete activities underpinning the economic dynamics of an industrial agglomeration. Over a two week period, data was collected by employing the geo-locative capabilities of Foursquare, a social media application, to record every movement of fashion workers employed at fashion design firms located both inside and outside the geographical boundaries of New York City’s Garment District. This unique method of studying worker activity exposed the day-to-day dynamics of an industrial district with a precision thus far undocumented in literature. Our work suggests that having access to the cluster provides almost the same agglomeration economies as residing within its borders.

## Introduction

From the Industrial Revolution to the present day, both theoretical and empirical work suggests that industrial clustering underpins prosperity and economic development on an urban, regional and national scale [1]–[4]. Over one hundred years later, Alfred Marshall’s conception of the industrial district remains central to modern economic principles [5]. Jane Jacobs’s seminal writings on the economies of cities put forth the concept of “new work” as enabled by external economies generated by the diversity and density of uses and people clustered on the same city blocks [6], [7]. Early motivations for proximity revolved around scale economies in production, high transport costs, and access to labor. Yet despite major changes in the technology of production, information communication technologies and the globalization of labor pools, industrial clustering persists. Four broad explanations underpin the importance of industrial agglomerations: localized knowledge-flows [8]–[13], external economies and cumulative advantage [14]–[22], spinoffs and entrepreneurship [23]–[25] and social relationships [26]–[30]. Longstanding studies suggest that agglomeration economies may play a role in economic development but how and why is still largely up for debate. To date, the relationship between the wider developmental effects of agglomeration economies and the mechanics of how this clustering operates spatially on a minute-to-minute, day-by-day level has not been quantified. This void can largely be explained by the inability to capture precise real-time data at a fine scale. These data limitations have hindered our ability to identify exactly how external economies or the benefits of proximity play out specifically across space and time. Understanding the mechanics of agglomeration economies could have profound outcomes for local and national development policy. The ability to jumpstart blighted urban economies from Detroit to Manchester, may hinge on understanding the daily dynamics that promote economic growth.

We use a unique dataset to study a world famous industrial agglomeration, the New York City Garment District. Previous research on this industrial cluster has identified its agglomeration economies as a source of innovation [31] and the networks that might explain its robustness despite ongoing contraction [*32*]. To capture the day-to-day functioning of the district, we use cell phone data and a social media application, FourSquare, to study the real time movements of garment industry design workers during their work day. We compare the movements of two groups of garment designers. In the first instance, we study the work activity of designers with firms located within the designated Garment District industrial cluster, geographically bounded by the New York City’s Fashion Center BID. The business location of fashion designers categorized as being outside the BID had a range of distances from the BID itself. They were not simply located just beyond the BID’s borders but in places as far and near as Brooklyn, Westchester County, Lower Manhattan and Queens. Although an administrative unit the Fashion BID was used as the boundary because while larger than the Garment District Manufacturing area, an analysis of garment related businesses showed the majority fell within the BID. Table 1 shows the number of apparel related establishments in the New York Metropolitan Statistical Area compared with those establishments in the zip code of New York City’s Fashion BID, it is clear that this small zip code comprises a large proportion of the industry. In the second group, we study designers whose firms are located outside of the industrial cluster but within the larger confines of the New York City Metropolitan Statistical Area (NY MSA). North American Industry Codes (NAICS) were used to determine these establishment numbers from the County Business Pattern data set 2011. The proper NAICS codes were identified from a previous study of the fashion industry performed by Williams & Currid-Halkett 2011, see Table S1 for NAICS codes [33]. The purpose of looking at firms located both inside and outside of this BID is to understand the extent to which spatial proximity matters in order to accrue the benefits of agglomeration economies.

**Table 1:** Number of Fashion Business Establishments: County Business Pattern Data 2011.

	In New York –New Jersey-LongIsland MSA	In Fashion Bid Zip Code (10018)	% in Fashion BID Zip Code (10018)
**Fashion Designers**	576	105	18.23%
**Wholesale**	7,128	1,119	15.70%
**Supplier**	1,344	275	20.46%
**Manufacturing**	1,473	399	27.09%

See Table S1 for North American Industry Codes (NAICS) used to come up with these establishment numbers. These NAICS codes were identified from Williams & Currid-Halkett 2011.

The data limitations of previous agglomeration studies may be overcome by the development of smart phone technologies that allow us to record individualized user data by place (latitude and longitude) and time. The use of smart phones for social research is emerging. Early examples of using programs on mobile devices to generate geo-located data, such as ContextPhone, were usually phone specific and cumbersome to modify stalling the use of mobile devices for data collection on social phenomenon [34]–[37]. Recently there has been a renewed interest in using cell phones for social research because some of the barriers have been removed. Lane et al. attribute four major changes in mobile computing that account for this shift. 1) sensors such as GPS units, microphones, light meters and accelerometers are embedded in most phones making data collection easier. 2) the operating systems and programming languages of cell phones are more open making it easier to program for them. 3) Cell phone vendors have applications stores that make it easier to download research driven programs. 4) The ability of mobile phones to send data to the cloud allows access to data quickly and without hindrance to study participants. (36) The renewed interest in using these devices has generated social/environmental research projects that use these mobile programs to track/collect GPS or cell phone tower trace information of study participants as they move through the urban environment. [38]–[39].

One of the biggest issues for using GPS trace data in cities is GPS positional error created by interference of satellite signals due to building blockage. Several studies show that GPS positions records can be off up to a few blocks which can be problematic in studies where highly precise information is needed. Researchers often employ cell phone tower triangulation and WI-FI positioning methods to enhance the location information collected on cell phones [40]–[41]. Researcher’s also use learning algorithms to help correct for error in GPS positions in areas where there are not as much interference from buildings and the positional error from the GPS can be more easily determine. [42] Social Media applications that record the locations of venues, such as Facebook and Foursquare, resolves issues related to GPS positional error by giving a static geo-location to venues users check-in to.

The static geo-positioning of social media applications and the ability to deploy user interfaces across various cell phone models and operating systems makes them useful tools for collecting data from diverse users. Some research has been done to understand how the geo-location capabilities of social media can be used to better understand place, but few studies have addressed how social media tools can be appropriated to generate data sets for economic and geographic research [43]–[48]. In this study we illustrate the possibilities of using mobile devices to analyze spatial patterns and movements within an industrial cluster at a scale and accuracy that has not previously been obtained.

## Materials and Methods

### Ethics Statement

This study was approved by the Internal Review board of Columbia University. The author was a Professor of the Columbia University at the time of the study. All Human participants of this project provided informed written consent according to the procedures of the Internal Review Board of Columbia University. During the approval period, all subjects enrolled provided voluntary informed consent to participate in the study and signed a copy of the appropriate stamped consent document(s). A copy of the consent document(s) was given to the subjects for their record. The requirement to obtain documentation of consent from subjects who will be consented by telephone has been approved in accordance with 45 C.F.R. § 46.117(c). The authors did not perform research for this project outside their country of residence.

### Smart Phones for Data Collection

FourSquare is a location based urban game available for smart phones by which players geographically register their current activity and share this information with other users. FourSquare users download the application onto their smart phones so that they can “check-in” to locations across the city. “Checking-in” to locations allows users to win prizes such as being a “Mayor” of their coffee shop by virtue of being the highest frequenting patron or, more monetary rewards such as getting a discount at a local bar. Foursquare saves this information as Latitude and Longitude records which can be retrieved using the program Application Programming Interface (API).

Participants of this study used Foursquare on their mobile phones to “check-in” to every location they visited during the work day during an “average” two week period, July 18^th^–July 29^th^ 2011. In order to capture the quotidian activities of fashion firms, we picked a relatively normal two week period during the year (rather than Fashion Week or right before designs go to production). The Latitude and Longitude location of every check-in was saved in Foursquare’s system and the information was downloaded through Foursquares’ API. Code was developed to interact with the API in order to download each participant’s activity log from the Foursquare database. This program provided a geo-referenced time dairy of the work they did and the businesses they went to over the course of the study. The result produced a highly accurate dataset detailing every movement of the participants of the study, therefore giving us a window into the precise interworking of the garment industry at an unprecedented scale.

The research focused on tracking the spatial behaviors of one segment of the garment industry, Fashion Designers. This sector was chosen because design firms use a cross section of the garment industry businesses to design and sell their products. Designers recruited from inside and outside the BID included well-known fashion design firms with larger staff, recent fashion school graduates, and fashion designers with start-up businesses. Overall, 77 fashion designers from 34 different design firms participated in the study. It should be noted that 100 fashion designers were recruited and agreed to participate in the study, however 77 designers actually actively engaged in the study once it started. The study sample represented roughly 3.5% of the fashion design employees and 6% of the fashion design firms in New York-Northern New Jersey-Long Island Metropolitan Statistical Area in 2011, using NAICS code 541490 for fashion designers in the County Business Pattern Data [49]. Each designer was categorized by size and type, Small Designers, Mid-Level Designers, Large Designers, and other Design/Manufacturers. The categorization was determined using employee counts, annual sales revenue, and year of establishment. Small Designers had a maximum of $300,000 in annual sales and on average 2 employees. Employees include partners and co-owners, but generally do not include interns. In comparison, Large Designers annual sales range from $1 million to $100 million and an average of 36 employees. Mid-Level designers fell in between with annual sales between $300,000 and $1 million, and on average 4 employees. Design/Manufacturers are businesses which have designers, but also specifically connect other designers to a larger network of manufacturers, suppliers and distributors, as a result the average annual sales for these firms is over $50,000,000 and over 50 employees. Each designer was also categorized by industry segment (Accessories, Manufacturing and Distribution, Men’s wear, Women and Men’s wear, and Alteration) and year business established. Table 2 has a breakdown of design firm categories and location.

**Table 2:** Participant Firms per Neighborhood.

*Type of Designer*	*FBID*	*Outside*	*TOTAL*
**Design/Manufacturers**	4		4
**Large**	2	3	5
**Mid-Level**	5	5	10
**Small**	1	14	15
**TOTAL**	**12**	**22**	**34**

### Data Interpretation: Network Analysis and Spatial Overlay to Understand Spatial Proximity

**Figure 1:**
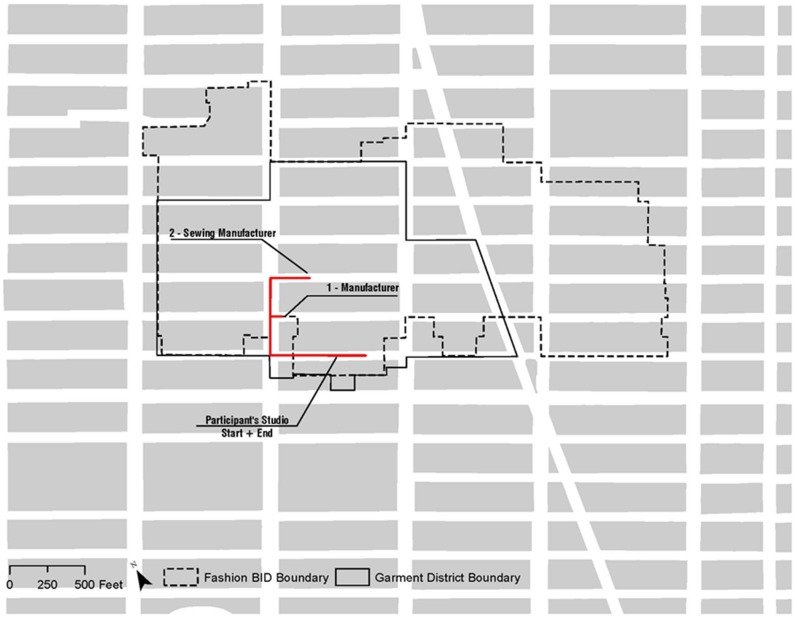
Example of one trip with 2 stops.

Participants of the study checked in using Foursquare over 2000 times making 508 unique trips during the course of the study (Figure 1 shows the spatial location of business visited). Check-in locations were established for every business that was visited by a fashion worker during the course of the study. In total, there were 287 unique businesses visited. Locations were categorized by industry type (Design, Manufacturing, Wholesale and Supply –see Table 3 & 4) and location type (Design, Education, Event Space, Financial, Hospitality, Manufacturer, Media, Retail, Service, Showroom, Social, Studio, Supply, Travel, Warehouse). Each check- in location c_j_ = (t_j_,l_j_) constituted a (time, location) pair, where time was measured accurately to the minute and locations were selected from latitude and longitude data coordinates established for Foursquare locations.

**Table 3:** Stops Made By Designers Located In The FBID.

	*Design*		*Manufacturing*	*Other*	*Supply*	*Wholesale*		*TOTAL*
**FBID**								
**Count of Stops**	17		290	15	97	41		460
**% of Stops**	3%		54%	3%	18%	8%		85%
**OUTSIDE THE FBID**								
**Count of stops**		5	45	5	8		18	81
**% of Stops**		1%	8%	1%	1%		3%	15%

Home studio locations excluded from stop totals as business movements were defined by those areas outside the studio.

**Table 4:** Trip Diversity Of Designers Located Outside The FBID.

	*Design*	*Manufacturing*	*Other*	*Supply*	*Wholesale*	*TOTAL*
**FBID**						
**Count of Stops**	3	171	3	77	28	282
	(2 designers)	(21 designers)	(3 designers)	(27 designers)	(9 designers)	
**% of Stops**	1%	44%	1%	20%	7%	73%
**OUTSIDE THE FBID**						
**Count of stops**	32	14	12	12	34	104
	(11 designers)	(8 designers)	(7 designers)	(8 designers)	(16 designers)	
**% of Stops**	8%	4%	3%	3%	9%	27%

Home studio locations excluded from stop totals as business movements were defined by those areas outside the studio.

* Note in cells above clarifies the number of participant designers that made those stops. It should be noted that there were a total of 44 design employees who were based outside the Fashion Center BID, which was defined as the agglomeration. There were 33 design employees located inside the Fashion Center BID.

**Figure 2:**
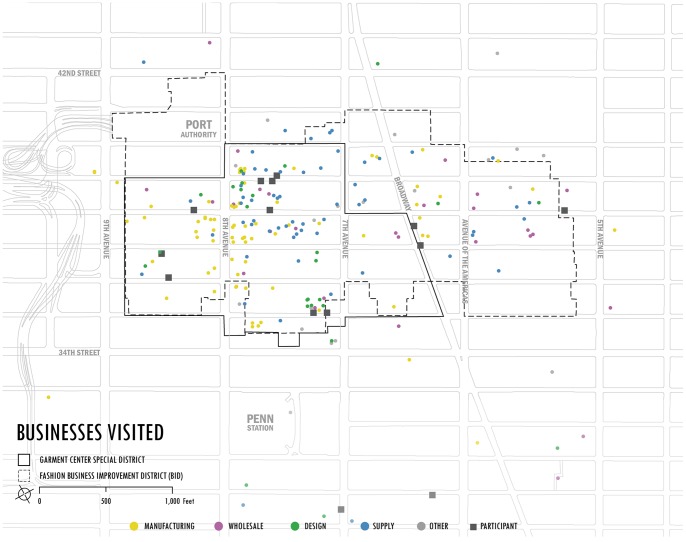
Zoom into the Fashion BID showing all the stops made categorized by type. Notice that there are more manufacturing firms located near 8^th^ avenue.

After categorizing the business locations, check-ins were also processed to identify unique trips. This was done in order to understand how the time and distance of business trips might help to illustrate possible advantages or disadvantages of locating inside and outside the industrial cluster. An individual trip T was defined as a participant leaving their studio or main office l_0_ and returning, i.e. any sequence of check-ins (c_a_ … c_c_) = ((t_a_,l_a_)… (t_b_,l_b_) … (t_c_,l_c_)) such that l_a_ = l_c_ = l_0_ and l_b_ ≠ l_0._ For example if an intern ran out to deliver a pattern to a manufacturing company and then stopped at another manufacturer to check on the progress of another article of clothing, and then headed back to the studio, the total route would be considered one trip (Figure 2). Over the course of the study there were a total of 508 trips with an overall average of 1.6 stops per trip.

This geo-registered latitude and longitude data helped to confirm participants’ location at a particular place at a given time, allowing us to overcome GPS noise interference issues typically associated with cities. [50]. GPS signal noise generated from building blockage can cause geo-registered locations on cell phones to be off from a few feet to a few city blocks, which can prevent researchers’ ability to identify actual routes taken by individuals using these points. The methods used in this study helped resolve this issue by having users check-in to known Foursquare places (venues) with established Latitude and Longitude information throughout the trip. Participants of the study checked-in to every business they visited during a business day. These confirmed check-ins helped to geo-locate participant paths and allowed us to develop routes from their string of geo-located business entries. The routing helped us to identify the timing of each segment of business trips and we used this time variables as a proxy for time and distance opportunity cost of performing business functions.

Network analysis modeling allowed us to pull out discrete distance and time variables of the trips designers made. These variables were used to understand how spatial patterns of trips played a role in how garment industry workers used the garment industrial district. Street routes for all business trips were generated from the network model. Routing was necessary because while we knew the actual start, end, and intermediate location stops of a trip, each route could not be determined from the GPS track data collected. This is because the GPS error was too high to estimate their position along the street network between one stop and the next. The shortest path routing algorithm was therefore used to estimate routes most likely taken. The route of each trip allowed us to determine the distance and time associated with getting to business, which we used as a proxy for the cost of performing garment related work. The sequence of locations making up each trip was modeled along New York City’s transport network using Dijkstra’s algorithm to determine the shortest path. The transport network was developed using New York City Department of City Planning’s LION network dataset employing intersection hierarchy, one way streets, and accessibility to junctions. Creating routes provided a way to estimate the distance and time it took to travel from one business to the next, by measuring the links along the route. Distance and time parameters were coded into each link used for analysis.

A spatial overlay algorithm was employed to determine whether a business locationexisted inside or outside the BID. This was done in order to understand to what extent traveling to businesses in the BID mattered to garment industry workers. A trip was considered to have entered the BID if any business stop along the trip was located in the BID. Each link L_i_ = ((t_i_,l_i_),(t_i+1_,l_i+1_) in a trip was also categorized as either a walking trip or a multi-model trip which meant participants either used a taxi, subway or train. Mode categorizations were determined by distance and time traveled across a link. Travel time for walking trips was estimated as 0.60 seconds per meter which is equivalent to a 16 minute mile stride. For other modes the actual link time t_i+1_ - t_i_ was recorded.

## Results and Discussion

### Economies of Scale: Organizing Around the Agglomeration

Our study demonstrates that the benefits of agglomeration are geographically nondiscriminatory within the broader garment industry, extending to workers located outside of the BID. Previous research argues that high levels of geographic clustering are necessary in knowledge-intensive industries [51]–[53]. Our results suggest that geographic location within the industrial cluster is not necessary for fashion firms to capture the garment district’s agglomeration economies. Overall the study shows that the BID is essential to those fashion designers working in the garment industry irrespective to whether their studio exists inside its boundaries. Regardless of geographic location inside or outside the BID, garment design workers use the different firms (e.g. design, supply, wholesale, manufacturing) within the agglomeration similarly, suggesting that capturing the benefits of the garment industry agglomeration is not dependent upon geographic location (Tables 3 & 4). All garment industry workers tend to disproportionately use firms within the BID over those located outside. Manufacturing represents the largest share of stops for all garment designers (54% for workers within the BID, 44% for those outside the BID ). Supply and wholesale firms are the next two most frequented types of firms (Tables 3 & 4). It should be noted that home locations were removed from analysis of overall stops as business movements were defined by those locations they went outside of the location of their studios.

**Figure 3:**
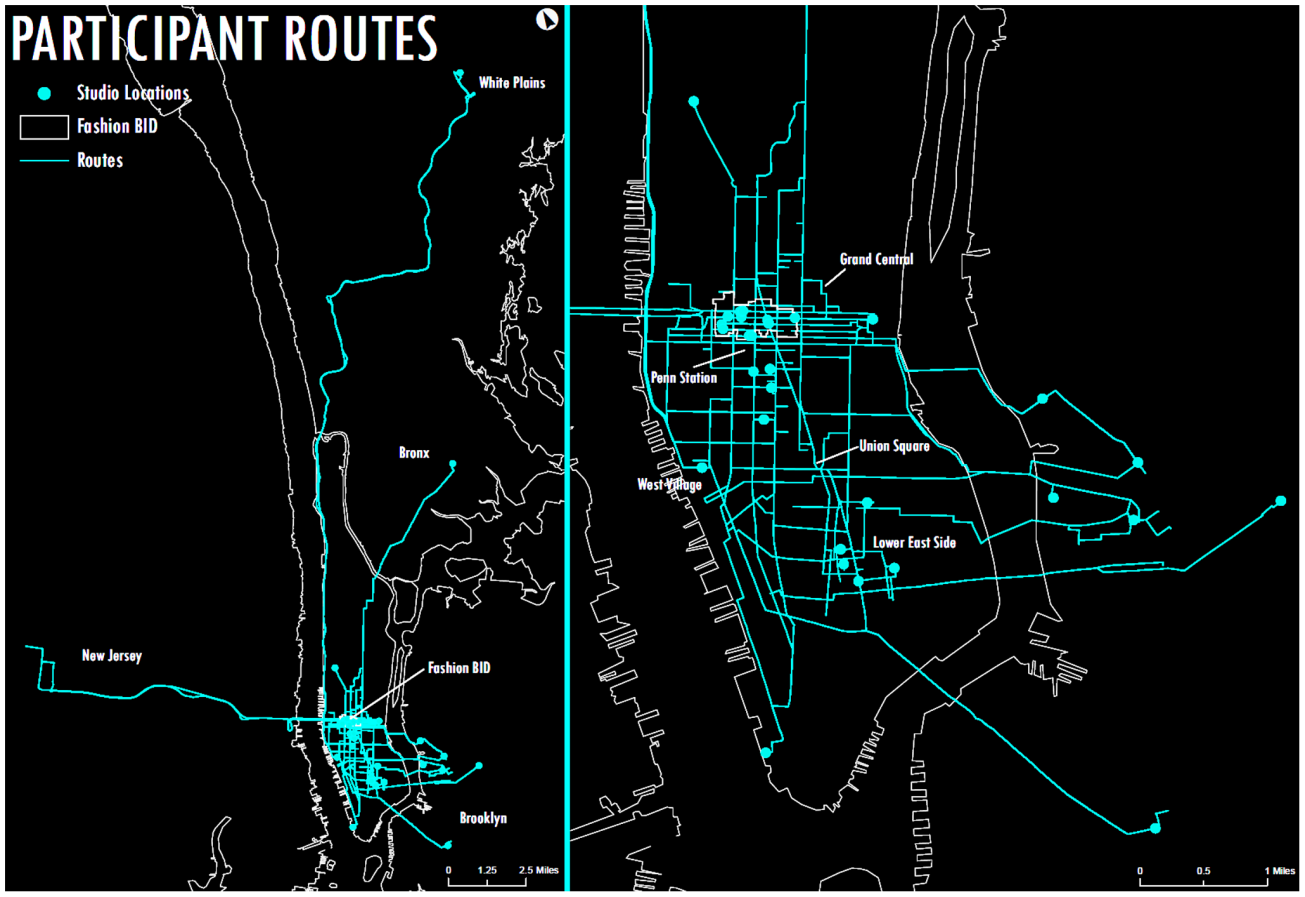
All trip routes made during the two week period.

Spatial movement data generated by the participants’ cell phones indicates that 77% of all trips by all designers (both those with firms located inside and outside the garment district) went into the BID at some point during the study, and 80% of all business-related were located in the BID. Figure 3 illustrates that the overwhelming amount of trips went into the Fashion BID during the two week period. As one might expect the agglomeration has increased benefits for those businesses inside its boundaries. Fashion design firms located within the BID make 85% of their business stops within its boundaries. Firms within the BID also tended to work within very close proximity to their studios: 91% of the trips were within a 15 minute walk of their firms.

Garment industry designers with firms located outside the BID interact within the agglomeration remarkably similarly to the designers located within the BID with one exception. While analysis of trip travel time showed that both workers located inside and outside the BID spent approximately the same amount of time performing trips overall those outside of the BID undertook an optimization strategy that involved bundling trips in order to compensate for the entry costs and travel time of being located farther away. Those designers with firms located within the BID were much less linear in performing business functions. During the two week period designers within the BID spend 100.8 minutes on an average of 9 trips versus 115.1 minutes for 4.8 trips for those designers located outside the BID. Overall, fashion designers inside and outside the BID spent the same time performing trips, but designers located within the district were able to make more trips within that same time, suggesting that the proximity allowed for idiosyncratic and ad hoc activities [*54*] crucial to fashion design work [*55*]. The choice of designers to locate firms outside of the BID may be linked to trade-offs in preferences between immediate proximity to resources and real estate prices, which are much higher in midtown Manhattan than in the boroughs where some designers choose to locate instead. More generally, we believe it is possible that the overall efficiencies are equal, with the farther away designers substituting fewer trips with more stops for more trips with fewer stops but ultimately each group investing the same amount of time in attaining necessary resources.

**Figure 4:**
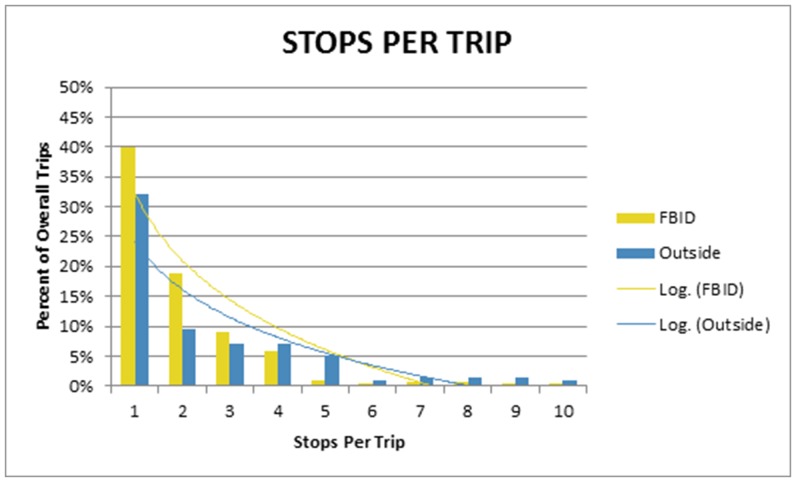
Looking all the trips of a 2 week period. Percentage of trips categorized by the number of stops exhibited.

Garment designers located outside the BID make an average of 15% more stops per trip to the BID. Fifty-one percent of trips made by outside designers are to the BID and of those trips 80% are chained together once a designer arrives. Consolidating trips to the district is also evidenced by the number of stops per trip garment workers outside the BID make versus those designers located within the BID: 18% versus 9%, respectively. (Figure 4).

### Agglomeration Dependence: Firm Size Matters

Firm size and type rather than geographical location appears to be the prominent explanation for how the agglomeration is used by the garment design firms. We find that large and mid-level firms with greater sale volume and employee count are most dependent on the manufacturers in the garment industry agglomeration while the smallest firms tend to be less reliant on manufacturing and prioritize other uses (e.g. research related to wholesale and supply products). Large and mid-level firms also exhibit similar patterns when it comes to using supply and wholesale business (Table 5 & 6). Smaller firms exhibit different patterns in their use of the district. They use supply (42%) and wholesale (29%) firms disproportionately more than manufacturing (25%)(Table 7).

**Table 5:** Large Designers Stops.

	*Design*			*Manufacturing*	*Other*		*Supply*	*Wholesale*		*TOTAL*
**FBID**										
**Stops**	7			347	6		64	16		440
**% in BID**	2%			79%	1%		15%	4%		100%
**OUTSIDE THE FBID**										
**Stops**		2	36		1	4			3	46
**% outside**		4%	78%		2%	9%			7%	100%

Home studio locations excluded from stop totals as business movements were defined by those areas outside the studio.

**Table 6:** Mid-Level Designers Stops.

	*Design*		*Manufacturing*	*Other*		*Supply*	*Wholesale*	*TOTAL*
**FBID**								
**Stops**	12		87	4		53	13	169
**% in BID**	7%		51%	2%		31%	8%	100%
**OUTSIDE THE FBID**								
**Stops**		0	10	1	2		11	24
**% outside**		0%	42%	4%	8%		46%	100%

Home studio locations excluded from stop totals as business movements were defined by those areas outside the studio.

**Table 7:** Small Designers Stops.

	*Design*			*Manufacturing*	*Other*		*Supply*	*Wholesale*		*TOTAL*
**FBID**										
**Stops**	1			23	2		39	27		92
**% in BID**	1%			25%	2%		42%	29%		100%
**OUTSIDE THE FBID**										
**Stops**		31	9		12	14			27	93
**% outside**		33%	10%		13%	15%			29%	100%

Home studio locations excluded from stop totals as business movements were defined by those areas outside the studio.

While small garment design firms do not use manufacturing firms as much as large and mid-level firms do, our results indicate that they nonetheless benefit from the external economies generated by the garment industry agglomeration, a finding that corroborates previous research on the industry [56]. From study exit interviews we found that this might be explained by the different production cycles and shorter production runs of the smaller firms. Several participants mentioned either not using manufacturers in the district or that they were not in a manufacturing cycle during the study. They noted that they did not need to release their fashion lines for spring, summer, fall and winter on the same timeline as larger designers as they were often not included in fashion week or made “just in time” fashion lines instead of lines that needed to be ordered month ahead of appearing in a store. These firms do not require the same relationships with multiple manufacturing businesses as a larger firm might. Forty-two percent of small firm activity is supply-related within the geographical boundaries of the BID, as compared to a large designer which performs only 15% of supplier activity within the BID.

### Flexible Industry: Allowing For “Just In Time” Fashion

**Figure 5:**
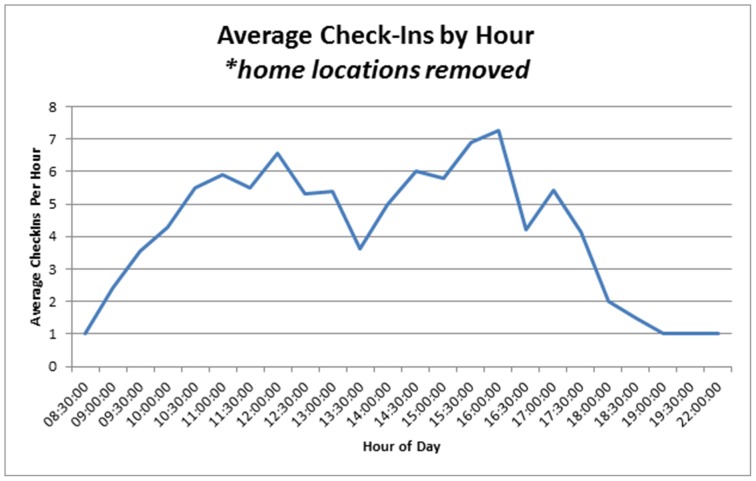
Average check-in count by hour. Home location or point of origin removed.

**Figure 6:**
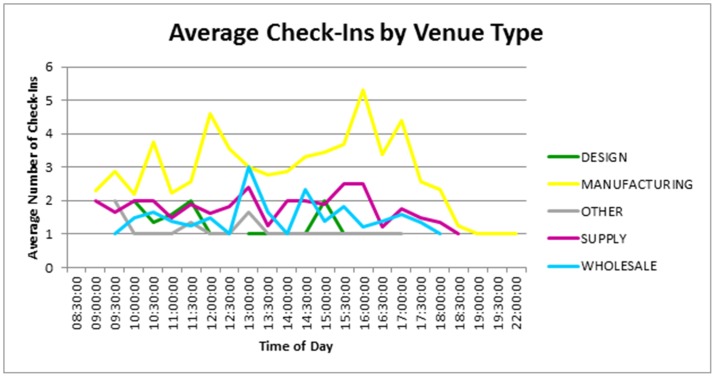
Average check-in count by hour, and check-in category. Home location removed. Manufacturing seems the most systematic with a drop in work during midday.

The spatial behavior of garment design workers suggests that the Garment District agglomeration allows for the chaos of the fashion design process and the “just-in-time” necessities of production in innovation-driven industries [57]–[62]. The garment designers do not exhibit set patterns to their spatial or temporal movements. Our aggregate study of the workers’ activity throughout the day demonstrates that while the workday is framed by regular hours (e.g. starting around 10 am and leaving at 6 pm with a lull in industrial activity from 1–2 pm for lunch) what the workers do hour by hour cannot be predicted (Figure 5). Put another way, on an average Wednesday, we do not find any regularity of industrial activity (e.g. they could be at a manufacturing firm, at the design house, or at a wholesaler at any given hour, regardless of firm size or geographical location of firm) (Figure 6). We offer two explanations for this observation. First, through the lens of one of the most innovation-driven industrial sectors, we are given insight into the chaotic and at times unsystematic nature of the creative process and how these dynamics are spatially manifested [63]. Second, the agglomeration’s most important contribution may be that it enables and facilitates the randomness and spontaneity of the innovation process by providing resources in an easy and accessible way such that these workers are able to optimize their workdays as directed by the creative process of garment design rather than by routine or limited to access resources [64], [65].

### Conclusions

This research set out to understand the micro level time and space interworking of the agglomeration economies within an industrial district. By developing a unique method of studying worker activity, our study of the New York City garment industry exposes the day-to-day dynamics of an industrial district with a precision thus far undocumented in the extant literature. This research produced several findings which buck conventional wisdom regarding the nature of agglomeration economies. Overall, the research indicates that agglomeration economies are important to the economic development of the garment industry but that the spillovers generated are captured by firms located substantially beyond the district’s immediate geographic boundaries. The robustness of the agglomeration corroborates previous work on the facility by which the garment industry is able to assemble and reassemble itself despite overall global shocks to the industry [*66*]. Taken together, the studies articulate some of the attributes that may explain the garment district’s long term resilience as an industrial district. Our findings highlight possible policy trajectories for development, indicating that discrete place-specific economic development may actually have a wide ranging effect on an industry across the region, whereby agglomeration economies do not operate as members-only benefits accrued only to those spatially proximate within the agglomeration. While by their very nature, agglomeration economies remain in situ, access is not restricted. From an economic development perspective, location costs and cost-prohibitive real estate prices associated with industrial clustering can be circumvented. The research would suggest that economic development of an industry may be cultivated by encouraging firm location in less expensive areas that are still privy to the localization economies of the industrial cluster nearby. The research also demonstrates that social media and smart phones technologies are a significant resource for developing detailed data about real life industrial and social dynamics that may challenge our theoretical assumptions about social and economic organization [*67]*, [68]. Our study offers new methodologies and data for understanding economic geography and industrial spatial dynamics on a micro scale never before achieved. Research of other industrial clusters would help to expose whether the patterns of regional economic benefits found within New York City’s garment industry exist within other agglomeration economies.

## Supporting Information

Figure S1 **Daily activities of Fashion Designer who participated in the study centered on locations in Lower/Middle Manhattan.**
(TIF)

Table S1 **NAICS Codes used for fashion industry definitions in** **Table 1** **.**
(DOCX)

Movie S1 **Animation of trips, routes, and stops that all designers visited as part of the study.**
(MP4)
