# Correction: Industry in Motion: Using Smart Phones to Explore the Spatial Network of the Garment Industry in New York City

**DOI:** 10.1371/journal.pone.0098832

**Published:** 2014-05-20

**Authors:** The PLOS ONE Staff

There are errors in the Author Contributions. The correct contributions are: Conceived and designed the experiments: SW EC. Analyzed the data: SW EC. Contributed reagents/materials/analysis tools: SW EC. Wrote the paper: SW EC.
