# Supplementary figures and images for: Industry in Motion: Using Smart Phones to Explore the Spatial Network of the Garment Industry in New York City
Source: PLoS One. 2014 Feb 5;9(2):e86165. doi: 10.1371/journal.pone.0086165 (PMC3914789; doi:10.1371/journal.pone.0086165)

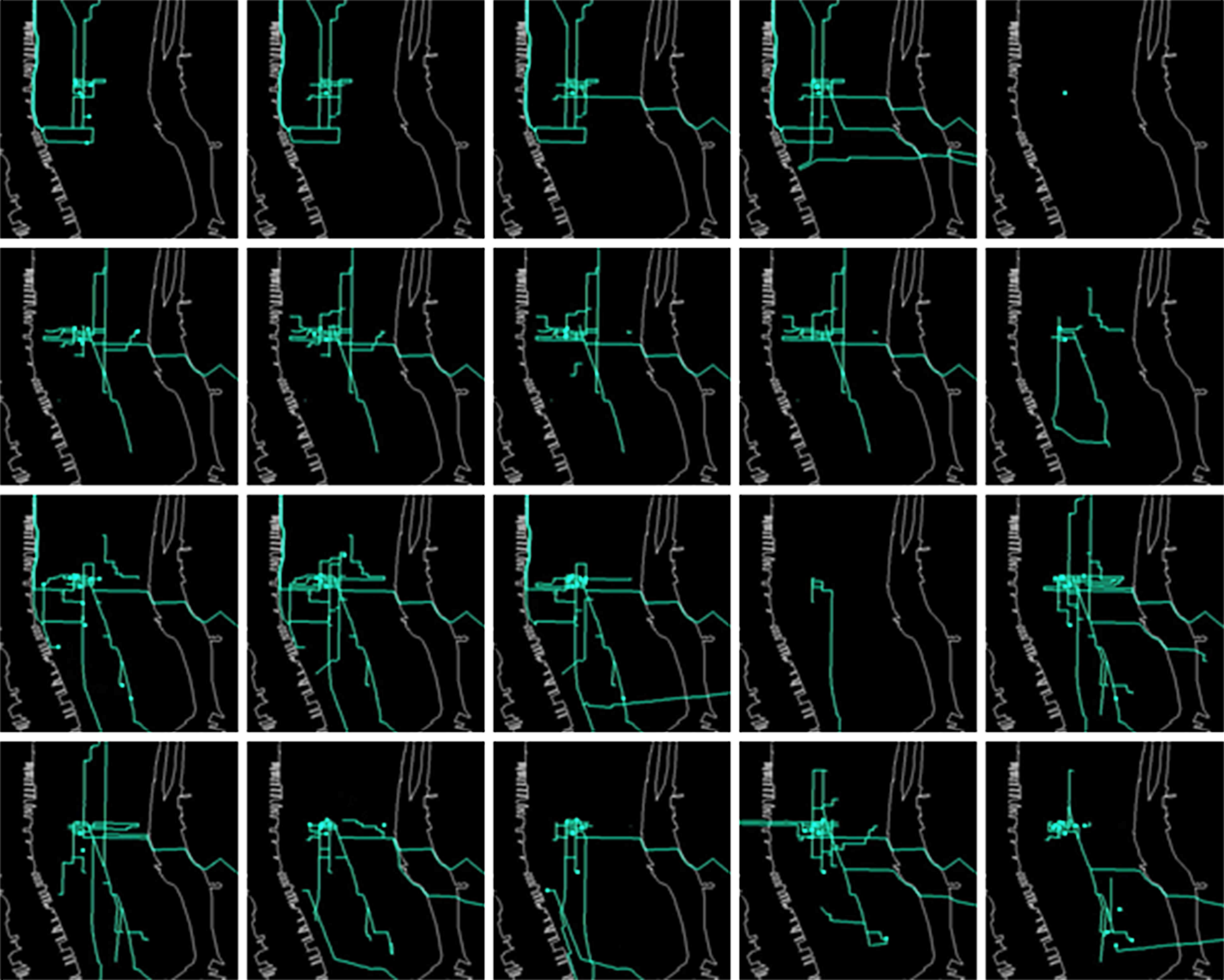

Supplement: Figure S1 — Daily activities of Fashion Designer who participated in the study centered on locations in Lower/Middle Manhattan. (TIF) [file pone.0086165.s001.tif]
