# Supplementary material for: Industry in Motion: Using Smart Phones to Explore the Spatial Network of the Garment Industry in New York City
Source: PLoS One. 2014 Feb 5;9(2):e86165. doi: 10.1371/journal.pone.0086165 (PMC3914789; doi:10.1371/journal.pone.0086165)
Supplement: Table S1 — NAICS Codes used for fashion industry definitions in Table 1 . (DOCX) [file pone.0086165.s002.docx]

| **NAICS Codes Used for Industry Definitions from the 2011 County Business Patterns** | |
| --- | --- |
|  |  |
| **Fashion Designers** |  |
| Other specialized design services | 541490 |
|  |  |
| **Wholesale** |  |
| Men’s and boys’ clothing and furnishings wholesalers | 424320 |
| Women’s, children’s and infants’ clothing and accessories merchant wholesalers | 424330 |
| Jewelry, watch, precious stone and precious metal merchant wholesalers | 423940 |
| Home furnishing merchant wholesalers | 423220 |
|  |  |
| **Supplier** |  |
| Textile mills | 313 |
| Textile mill products | 314 |
| Piece goods, notions and other dry goods merchant wholesalers | 424310 |
|  |  |
| **Manufacturing** |  |
| Apparel manufacturing | 315 |
| Leather and allied product manufacturing | 316 |
| Costume jewelry and novelty manufacturing | 339914 |
| Fastener, button, needle, and pin manufacturing | 339993 |
